# A case of IgG4-related disease coexisted with rectal cancer

**DOI:** 10.1186/s40792-015-0120-7

**Published:** 2015-11-24

**Authors:** Takeshi Tsuchiya, Takahiro Yagi, Mitsuo Tsukamoto, Yoshihisa Fukushima, Ryu Shimada, Keisuke Nakamura, Shoichi Fujii, Keijiro Nozawa, Keiji Matsuda, Yoshinao Kikuchi, Koji Saito, Yojiro Hashiguchi

**Affiliations:** The Department of Surgery, Teikyo University School of Medicine, 2-11-1 Kaga, Itabashi-ku, Tokyo 173-0003 Japan; The Department of Pathology, Teikyo University School of Medicine, 2-11-1 Kaga, Itabashi-ku, Tokyo 173-0003 Japan

**Keywords:** IgG4-related disease, Rectal cancer, Sclerosing cholangitis

## Abstract

A 67-year-old man was referred to our hospital with suspicion of rectal tumor, hilar tumor, and urinary tumor. Colonoscopic findings were intermittent nodular lesions with redness which were atypical to primary rectal cancer. Endoscopic retrograde cholangiopancreatography showed narrowing of the bilateral intrahepatic bile duct. However, the findings were improved 1 month later. Blood biochemistry showed high level of serum IgG4 up to 1140 mg/dl. The patient matched to comprehensive diagnostic criteria for IgG4-related disease as a possible diagnostic case. Laparoscopic low anterior resection with creation of ileostomy was performed for rectal cancer. Histological findings revealed cancer cells spread horizontally at submucosal layer and subserosal layer. There was marked infiltration of the plasma cells and lymphocytes at tumor stroma, and more than half of the plasma cells were positive for IgG4. After surgery, the level of serum IgG4 was decreased to 597 mg/dl. Although the association with IgG4-related disease and colorectal disease is unclear, the tumor progression was atypical for rectal cancer. Some report that the disease may rise up the risk of a malignant disease. It is necessary to perform systemic examination keeping in mind for concurrence of malignancy.

## Background

IgG4-related disease is the notion which involves enlargement, tumor, nodule, and thickening lesion in various kinds of systemic organs. It is characterized by marked infiltration of lymphocytes and IgG4-positive plasma cells and fibrosis [1]. Autoimmune pancreatitis and sclerosing cholangitis are well-known digestive diseases among IgG4-related diseases. The association between IgG4-related disease and colorectal disease is unclear. There are only a few reports about concurrence of these diseases. It is also uncertain whether IgG4-related disease is a risk factor of malignant tumors or not. In this study, we report a case of IgG4-related disease coexisted with rectal cancer.

## Case presentation

**Fig. 1:**
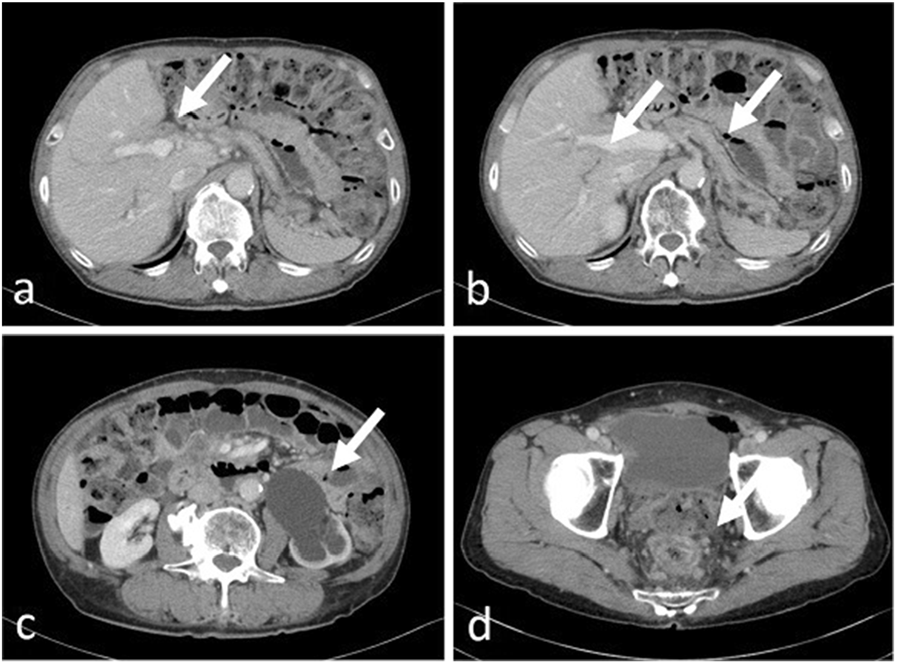
CT findings. Thickening of the hilar bile duct (**a**: *arrow*) and dilatation of the intrahepatic bile duct (**b**: *arrow*) were shown. The pancreas was not enlarged (**b**: *arrow*). Dilatation of the left renal pelvis (**c**: *arrow*) and thickening of the rectal wall (**d**: *arrow*) were shown

**Fig. 2:**
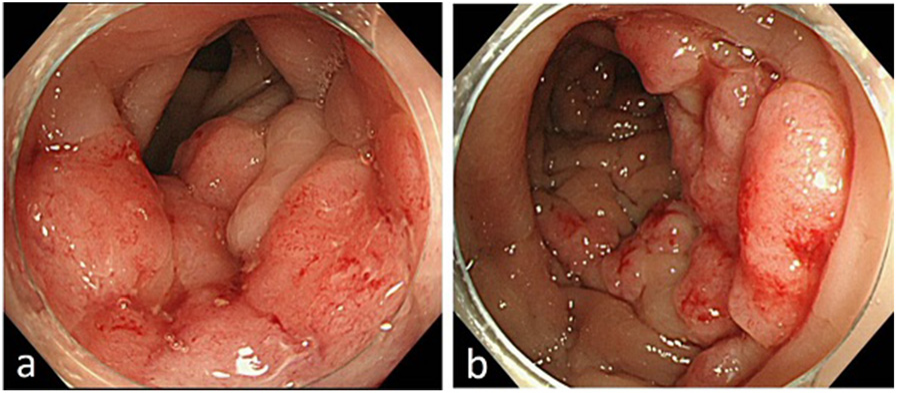
**a**, **b** Colonoscopic findings. Intermittent nodular lesions with redness in the rectum were seen

**Fig. 3:**
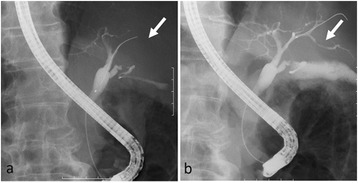
ERCP findings. **a** Narrowing of the bilateral intrahepatic bile duct was shown. **b** One month later, the narrowing of the right intrahepatic bile duct improved

**Fig. 4:**
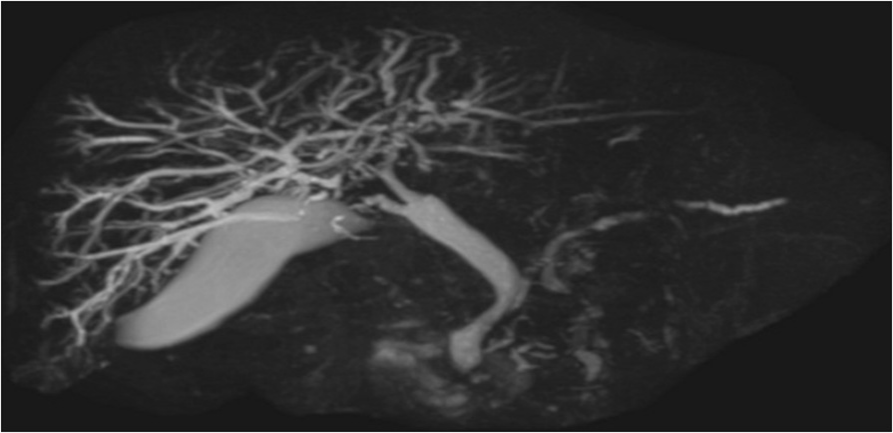
MRCP findings. Narrowing of the bilateral intrahepatic bile duct and the main pancreatic duct was shown

**Fig. 5:**
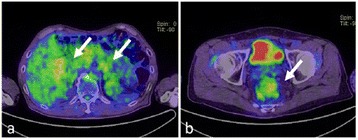
PET findings. Accumulation to the hilar bile duct (**a**: *arrow*), pancreatic body and tail (**a**: *arrow*), and rectum (**b**: *arrow*) was shown

**Fig. 6:**
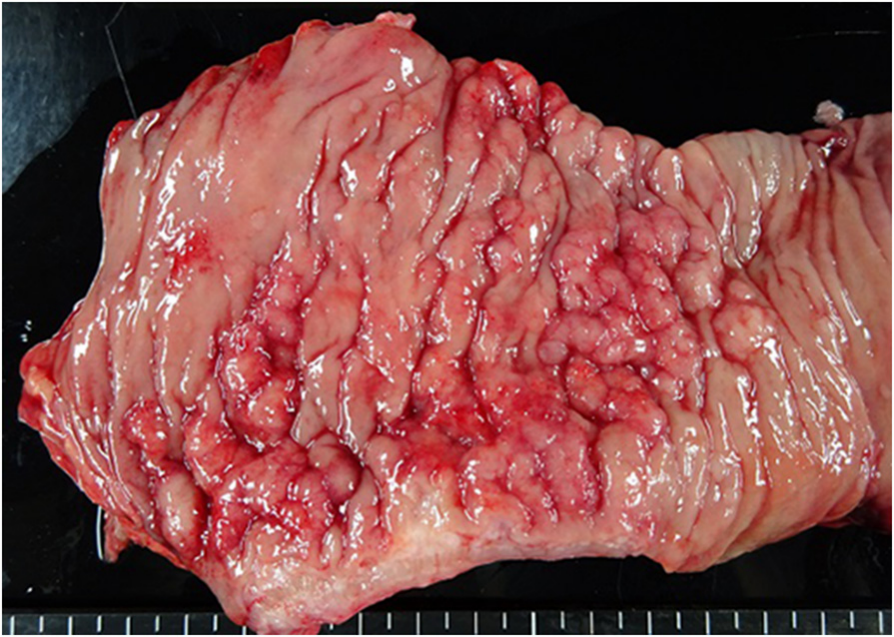
The resected specimen. Multiple nodular lesions in the rectum were shown

**Fig. 7:**
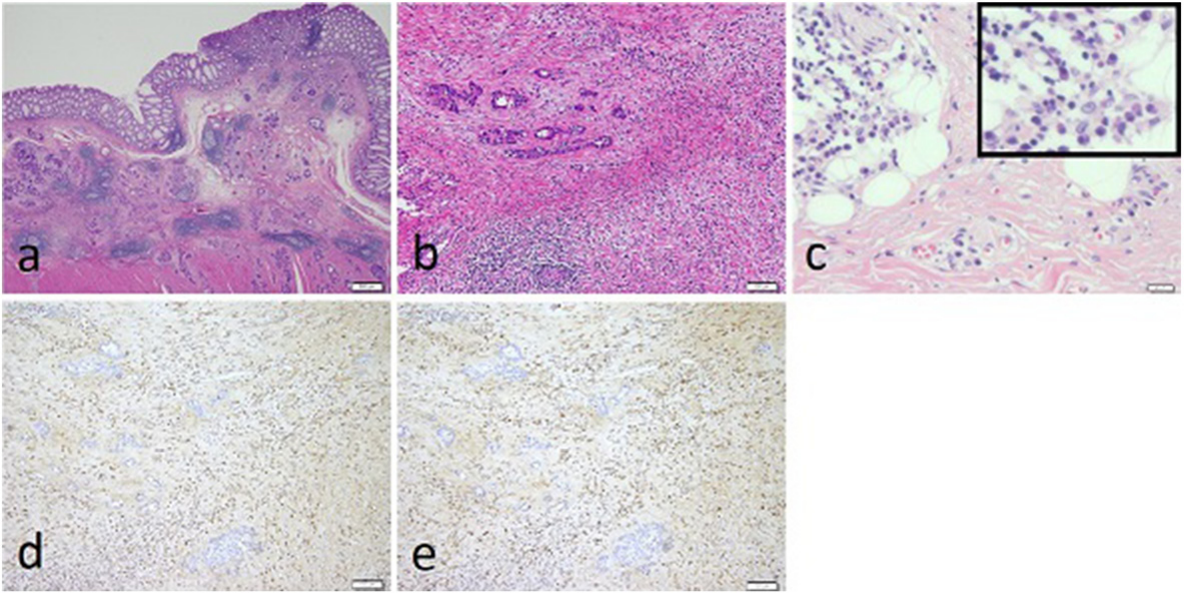
Histological findings. **a** Hematoxylin-eosin staining (×20), cancer cells spread horizontally at submucosal layer. **b** Hematoxylin-eosin staining (×100), marked infiltration of the plasma cells and lymphocytes was shown at tumor stroma. **c** Hematoxylin-eosin staining (×400), infiltration of plasma cells and fibrosis were shown in the retroperitoneal tissue. **d** IgG-immunostaining (×100) and **e** IgG4-immunostaining (×100), more than half of the infiltrating plasma cells were positive for IgG4

A 67-year-old man was referred to our hospital with suspicion of rectal tumor, hilar tumor, and urinary tumor. He had hyper urine acid and diabetes mellitus. There were no abnormal physical findings. Blood biochemistry showed slight increase of the CEA, CA19-9, and SPAN-1 levels to 6.7 ng/ml, 45.7 U/ml, and 33 U/ml, respectively. Computed tomography (CT) showed thickening of the hilar bile duct, dilatation of the bilateral intrahepatic bile duct, swelling of the para aortic lymph node, dilatation of the left renal pelvis, and thickening of the rectal wall. The pancreas was not enlarged (Fig. 1). Colonoscopy revealed intermittent nodular lesions with redness in the rectum (Fig. 2). They were atypical to primary rectal cancer. Histopathological examination suggested a well-differentiated adenocarcinoma. At this point, we suspected metastatic rectal cancer as diagnosis and conducted systemic examination continuously. Endoscopic retrograde cholangiopancreatography (ERCP) was performed. It showed narrowing of the bilateral intrahepatic bile duct, though biopsy of the bile duct was negative for malignant tumor (Fig. 3a). ERCP was reexamined 1 month later. The narrowing of the right intrahepatic bile duct improved except for slight segmental stricture of the peripheral bile duct (Fig. 3b). Brushing cytology of the bile duct was negative for malignant tumor. Magnetic resonance cholangiopancreatography (MRCP) showed narrowing of the bilateral intrahepatic bile duct and the main pancreatic duct (Fig. 4). Positron emission tomography (PET) showed accumulation to the hilar bile duct, pancreatic body and tail, rectum and lymph nodes of the pulmonary hilar lesion, axilla, and para aorta (Fig. 5). We considered possibility of the IgG4-related disease and measured the level of serum IgG4. Blood biochemistry showed high level of serum IgG4 up to 1140 mg/dl. The patient matched to the comprehensive diagnostic criteria for IgG4-related disease as a possible diagnostic case. He was finally diagnosed with rectal cancer with IgG4-related disease (sclerosing cholangitis and retroperitoneal fibrosis leading to hydronephrosis were suspected). We performed laparoscopic low anterior resection of the rectum with creation of ileostomy for rectal cancer. In the intraoperative findings, there was retroperitoneal fibrosis. The periarterial tissue, especially anterior tissue of the abdominal aorta, was hard. The tissue around the left ureter crossing the common iliac artery was also hard, and caliber change of the ureter was seen at the area. No evidence of urinary tumor was seen. The mesorectum was thick and edematous. The lateral tissue of rectum was also hard. The resected specimen revealed multiple nodular lesions in the rectum (Fig. 6). Histologically, moderately differentiated adenocarcinoma cells were infiltrating through the rectal wall. Cancer cells spread horizontally at submucosal layer and subserosal layer. Massive lymph nodes involvement, lymphatic invasion, venous invasion, and perineural invasion were also revealed. There was marked infiltration of the plasma cells and lymphocytes at tumor stroma. In addition, infiltration of inflammatory cells containing plasma cells and fibrosis were also seen in the retroperitoneal tissue apart from the cancer lesion. Immunohistochemistrical findings revealed that more than half of the plasma cells infiltrating connective tissue were positive for IgG4 (Fig. 7). The final stage of the rectal cancer was T3N2bM0 stage IIIC according to the TNM classification (UICC 7th edition). The postoperative course was uneventful except for paralytic bowel obstruction. For adjuvant therapy, the patient received modified FOLFOX6. After surgery, the level of serum IgG4 decreased to 597 mg/dl.

### Discussion

IgG4-related disease is a relatively new disease entity transmitted to the world from Japan. Comprehensive diagnostic criterion for IgG4-related disease was advocated in 2012 [1]. It was composed of three points: (1) clinically, existence of enlargement, tumor, nodule, and thickening lesion in various kinds of organs; (2) blood chemistrically, elevated serum IgG4 concentration >135 mg/dl; and (3) pathologically, marked infiltration of lymphocytes and plasma cells and fibrosis, >40 % of IgG-positive plasma cells being IgG4 positive, and >10 cells/high-powered field being IgG4 positive. The disease includes autoimmune pancreatitis, sclerosing cholangitis, sialadenitis, interstitial pneumonitis, retroperitoneal fibrosis, etc. They have good response to steroid therapy in many cases.

In this case, thickening of the bile duct and elevated serum IgG4 concentration strongly suggested IgG4-related cholangitis. Infiltration of plasma cells and fibrosis in the retroperitoneal tissue also supported the existence of IgG4-related retroperitoneal fibrosis. Pathological findings of the rectal specimen showed that IgG4-positive plasma cells infiltrated to the connective tissue around rectal cancer. Some reported abundant infiltration of IgG4-positive plasma cells was revealed around malignant tumor in six of eight cases of malignant tumor with autoimmune pancreatitis [2]. On the other hand, moderate infiltration of them was revealed around gastric cancer in 2 of 40 cases of gastric cancer without autoimmune pancreatitis. It was indicated that abundant infiltration of IgG4-positive plasma cells around malignant tumor could be regarded as specific findings in malignant tumor with autoimmune pancreatitis. It was uncertain whether rectal cancer developed sequentially to IgG4-related rectal lesion or IgG4-positive plasma cells infiltrated according to rectal cancer progression.

Histological findings revealed that cancer cells mainly resided in submucosal layer and they partly spread to mucosal layer. The cluster of cancer cells was seen diffusely through the rectal wall. We speculated that the cancer progressed by mural metastasis, and this unique pattern of progression might be affected by infiltration of IgG4-positive plasma cells.

To assess the stage of cancer progression, PET was performed. Because the positive accumulation to the lesion of IgG4-related disease was also observed, it was difficult to distinguish cancer from the disease. In this case, systemic lymph node swelling and accumulation to them were shown by PET. We considered that it was due to IgG4-related disease because distribution of the swelling lymph nodes was not usual for rectal cancer. The patient received adjuvant chemotherapy (modified FOLFOX6) because the cancer was staged as stage IIIC.

Intraoperative findings showed sclerosis around the artery and stenosis of the ureter which indicated periarteritis and retroperitoneal fibrosis. These findings also supported to make diagnosis of IgG4-related disease.

Although sclerosing esophagitis, gastric ulcer, and polyps were reported as gastrointestinal tract lesion of IgG4-related disease [3–6], the association between IgG4-related disease and colorectal lesion was unclear. Just four cases reportedly showed thickening of the cecum, sigmoid colon, ileocecal region, and anus [7–9].

Recently, coexistence of IgG4-related disease and malignant tumor was reported [10–12]. The incidence of malignant tumor in patient of autoimmune pancreatitis was nearly three times higher than in normal population [2]. Only two cases of rectal cancer with IgG4-related disease were previously reported in the literature [13, 14]. In those cases, no infiltration of IgG4-positive plasma cells was seen in and around the rectal cancer. Thus, this is the first report of potent infiltration of IgG4-positive plasma cells to the connective tissue around rectal cancer in a patient with IgG4-related disease. The process of cancer development might be different among these cases. To elucidate the relation of IgG4-related disease and malignant tumor, it is necessary to accumulate more cases of these diseases.

## Conclusions

We experienced a case of IgG4-related disease with rectal cancer which had atypical progression. It is difficult to distinguish IgG4-related disease from malignancy. As the disease sometimes coexists with malignant disease of other organs, it is necessary to perform systemic examination keeping in mind for concurrence of malignancy.

## Consent

Written informed consent was obtained from the patient for publication of this case report and any accompanying images. A copy of the written consent is available for review by the Editor-in-Chief of this journal.

## Abbreviations

CT: computed tomography
ERCP: endoscopic retrograde cholangiopancreatography
MRCP: magnetic resonance cholangiopancreatography
PET: positron emission tomography
